# Histone H3 lysine 36 methylation affects temperature-induced alternative splicing and flowering in plants

**DOI:** 10.1186/s13059-017-1235-x

**Published:** 2017-06-01

**Authors:** A. Pajoro, E. Severing, G. C. Angenent, R. G. H. Immink

**Affiliations:** 1 Laboratory of Molecular Biology, Wageningen University and Research, 6708 PB Wageningen, The Netherlands; 2 Bioscience, Wageningen University and Research, 6708 PB Wageningen, The Netherlands; 3 Laboratory of Bioinformatics, Wageningen University and Research, 6708 PB Wageningen, The Netherlands; 4 Max Planck Institute for Plant Breeding Research, 50829 Köln, Germany

**Keywords:** Ambient temperature, Alternative splicing, Histone modification, H3K36me3, Flowering time, SDG8, Arabidopsis

## Abstract

**Background:**

Global warming severely affects flowering time and reproductive success of plants. Alternative splicing of pre-messenger RNA (mRNA) is an important mechanism underlying ambient temperature-controlled responses in plants, yet its regulation is poorly understood. An increase in temperature promotes changes in plant morphology as well as the transition from the vegetative to the reproductive phase in *Arabidopsis thaliana* via changes in splicing of key regulatory genes. Here we investigate whether a particular histone modification affects ambient temperature-induced alternative splicing and flowering time.

**Results:**

We use a genome-wide approach and perform RNA-sequencing (RNA-seq) analyses and histone H3 lysine 36 tri-methylation (H3K36me3) chromatin immunoprecipitation sequencing (ChIP-seq) in plants exposed to different ambient temperatures. Analysis and comparison of these datasets reveal that temperature-induced differentially spliced genes are enriched in H3K36me3. Moreover, we find that reduction of H3K36me3 deposition causes alteration in temperature-induced alternative splicing. We also show that plants with mutations in H3K36me3 writers, eraser, or readers have altered high ambient temperature-induced flowering.

**Conclusions:**

Our results show a key role for the histone mark H3K36me3 in splicing regulation and plant plasticity to fluctuating ambient temperature. Our findings open new perspectives for the breeding of crops that can better cope with environmental changes due to climate change.

**Electronic supplementary material:**

The online version of this article (doi:10.1186/s13059-017-1235-x) contains supplementary material, which is available to authorized users.

## Background

Plants sense changes in the environment and adapt their development accordingly. Temperature is one of the environmental signals that strongly affects plant development. For example, plants are able to adapt their organ shape in relation to the temperature they experience, a phenomenon called “thermo-morphogenesis” [1]. As such, elevated temperature promotes hypocotyl elongation and plants also show differences in leaf morphology depending on the growing temperature [2–5]. The circadian clock, the internal clock that confers an organism the innate ability to measure time, is also influenced by temperature [6–9]. Another important plant trait controlled by temperature is flowering time, the switch from the vegetative to the reproductive phase. An increase in ambient temperature promotes flowering in Arabidopsis and, consequently, at high ambient temperatures these plants flower earlier than at colder conditions [10–12].

A change in ambient temperature affects gene functioning at transcription level and at post-transcriptional level, e.g. through protein conformation and stability. Recently, it has been shown that ambient temperature affects phytochrome B (PHYB) inactivation during the night [13]. Moreover, protein stability can be influenced by temperature, for example the flowering time regulator short vegetative phase (SVP) is subject to faster protein degradation at higher temperature [14]. Finally, ambient temperature also affects alternative splicing [15]. Alternative splicing (AS) is the process that produces different mature messenger RNAs (mRNAs) from a single pre-mRNA [16]. AS is a rapid and adjustable process that allows a fast response to external stimuli and facilitates plasticity in plant development [17]. In plants, key regulators of various processes such as circadian clock, flowering time, and reproduction are regulated via temperature-induced AS [14, 15, 18–24]. Ambient temperature changes also affect splicing of AS regulators and thereby plant development [25, 26]. As such, AS was suggested to act as “molecular thermosensor” in plants [15]; however, how temperature can affect splicing of a specific subset of intron-containing genes is poorly understood. In higher eukaryotes, it has been shown that pre-mRNA splicing occurs co-transcriptionally in the nucleus. Thus, while RNA polymerase is synthesizing the pre-mRNA, the splicing machinery is recruited to perform its function [27]. The co-transcriptional nature of splicing allows a contribution of the chromatin landscape in splicing regulation [28–30]. In mammals, two models have been proposed to explain the interplay between chromatin landscape and AS: the kinetic model and the chromatin-adaptor model [31]. Evidence for both modes of action has been reported. According to the kinetic model, the chromatin landscape can influence the transcription elongation rate and thereby affects splicing. For example, histone acetylation allows opening of the chromatin leading to a fast elongation rate that promotes splice site skipping. To the contrary, silencing histone marks lead to a more compact chromatin and slow elongation rate allowing recognition of weak splicing sites [29]. In the chromatin-adaptor model, histone modifications are bound by readers able to recruit splicing factors. An example is the H3K36me3-MRG15-PTB chromatin-adaptor system. The H3K36me3 histone mark serves as an anchor for binding of the adaptor protein MRG15, which in turn interacts with the splicing regulator polypyrimidine tract-binding (PTB) recruiting it to the nascent pre-mRNA [32].

In plants, it is not known if a similar link between chromatin and splicing exists and whether such a mechanism would be ambient temperature sensitive. H3K36me3 was found to be prevalent in transcribed regions and therefore a function in transcriptional elongation has been postulated for this histone mark [33]. Here we investigated the potential contribution of this mark in the regulation of ambient temperature-induced AS and flowering time control in the model plant *Arabidopsis thaliana*.

## Results

### Identification of temperature-induced differentially spliced events

**Fig. 1:**
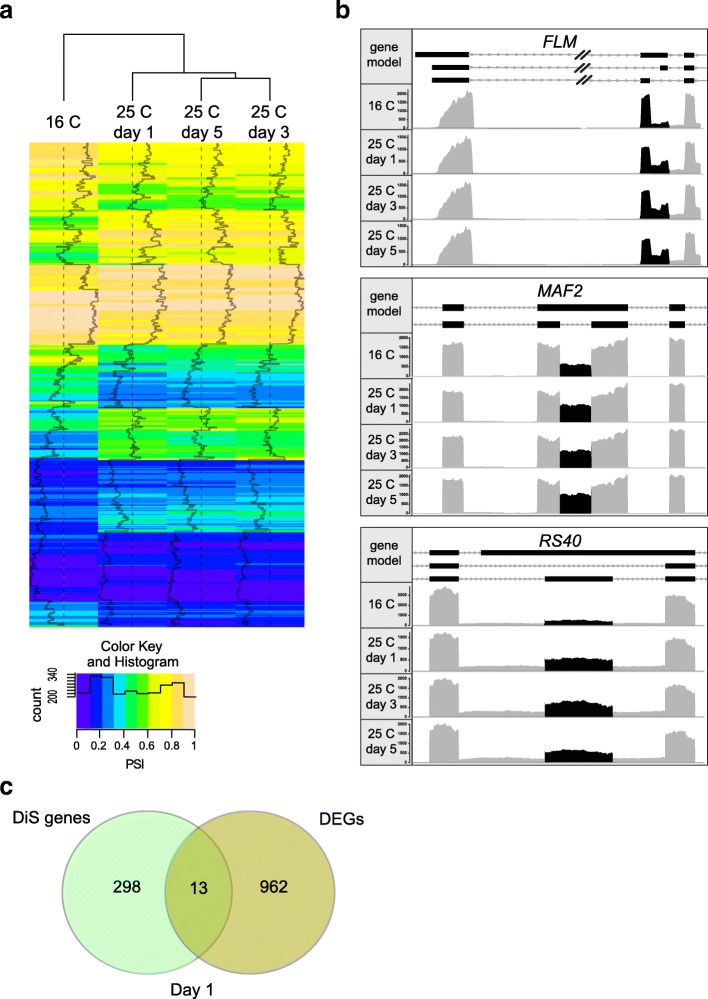
Dynamics of temperature-induced DiS events. **a** *Heat map* showing the percentage of spliced in (PSI) values obtained at each condition for significant DiS events. **b** Partial gene model depicting the detected DiS events of *FLOWERING LOCUS M* (*FLM*), *MADS AFFECTING FLOWERING 2* (*MAF2*), and *ARGININE/SERINE-RICH SPLICING FACTOR 40* (*RS40*), and corresponding plot area showing RNA-seq results. *Coverage plot* reads within the AS regions are shown in *black*, other reads are in *gray*. We detected a significant difference in IR events for *FLM* [35] and *MAF2* [34] and MXE events for *FLM* [21] and *RS40* upon the temperature change. **c** *Venn diagram* shows the overlap between DiS and totally differentially expressed genes (DEGs) one day upon the temperature change. DEGs are defined as genes with a change in expression of log2 Fold Change |1| and adjusted *p* value according to the BH method for controlling false discovery < 0.05

To study the proposed linkage between the H3K36me3 mark and ambient temperature-induced AS, we first identified transcriptome-wide differentially spliced (DiS) events upon a temperature change and investigated the temporal dynamics of this process. Arabidopsis plants were grown at 16 °C in short day conditions (8 h light-16 h dark) for five weeks and then transferred to 25 °C. With RNA sequencing (RNA-seq), we analyzed the changes in the transcriptome at one, three, and five days after the temperature change. This experimental setup allows to monitor initial changes in splicing pattern upon a temperature change and to investigate the stability of these effects. For each time point, we quantified AS setting one isoform as reference and then calculating the “Percentage of Spliced In” (PSI) for each type of event (intron retention [IR], mutually exclusive exon [MXE], exon skipping [ES], alternative 3′ site [A3], alternative 5′ site [A5]; Additional file 1: Table S1). We defined isoforms that present a significant difference in PSI (ΔPSI) between two conditions as temperature-induced DiS. In total, we identified 683 DiS events, derived from 511 distinct genes, involved in a plethora of biological processes (Additional file 1: Table S1, Fig. 1a, and Additional file 2: Figure S1A).

We found DiS events in genes previously reported to be subject to temperature-induced AS, such as in the flowering time regulators *FLOWERING LOCUS M* (*FLM*) [14, 21] and *MADS AFFECTING FLOWERING 2* (*MAF2*) [22, 34] (Additional file 2: Figure S1B) and the clock components *PSEUDO-RESPONSE REGULATOR 3* (*PRR3*) and *PRR7* [8, 18]. For *FLM*, we observed an increase in the retention of the second intron and a MXE event [14, 21, 35]. The retention of the second intron leads to a transcript targeted for degradation by nonsense-mediated mRNA decay (NMD) and as such decreased amount of functional protein at higher temperature [35], while the MXE event is supposed to result in the production of two proteins with opposite function in flowering time regulation [21]. The IR at *MAF2* locus generates an isoform with a premature stop codon that is most likely targeted for NMD and the increased abundance of this isoform at higher temperature leads to a decrease of the functional flowering time repressor [34]. *PRR3* and *PRR7* were shown previously to undergo AS upon a change to low temperature from 20 °C to 4 °C [18]. In our experiment, we see a change in retention of the third intron of *PRR7* as previously reported for a change to low temperature, as well as new events for *PRR7* and *PRR3* that appear to be specific to the increase in ambient temperature in our experimental setup (Additional file 1: Table S1).

Moreover, we found splicing regulators DiS upon the temperature change, such as *RS40* (Fig. 1b), *SC35-LIKE SPLICING FACTOR 33* (*SCL33*), *SERINE/ARGININE-RICH PROTEIN SPLICING FACTOR 34* (*SR34*), and the U2 snRNP auxilliary factors U2AF65A and U2AF65B.

Additionally, we also identify novel temperature-induced splicing events in master regulators of various biological processes, such as the flowering time regulator *SQUAMOSA PROMOTER BINDING PROTEIN-LIKE 2* (*SPL2*) [36] and *MODIFIER OF SNC 1* (*MOS1*) [37], the regulators of auxin signaling *AUXIN RESPONSE FACTOR 2* (*ARF2*) [38] and *PIN-FORMED 7* (*PIN7*) [39], and the chromatin remodelers *VIN3-LIKE 2* (*VIL2*) [40] and *SPLAYED* (*SYD*) [41–43] (Fig. 1b and Additional file 1: Table S1).

The majority of the genes show DiS within one day upon the temperature change and after that their PSI remained stable over time (Fig. 1a, b), revealing a response to a change in ambient temperature within 24 h. Surprisingly, we found only a small overlap (13 genes) between differentially expressed genes (DEGs) (Additional file 3: Table S2) and DiS genes (Fig. 1c), indicating a different basis for the effect of ambient temperature on these two molecular processes.

In conclusion, a change in ambient temperature triggers fast and stable changes in splicing of many key regulatory genes, including various flowering time regulators.

### DiS genes are enriched in H3K36me3 modification

**Fig. 2:**
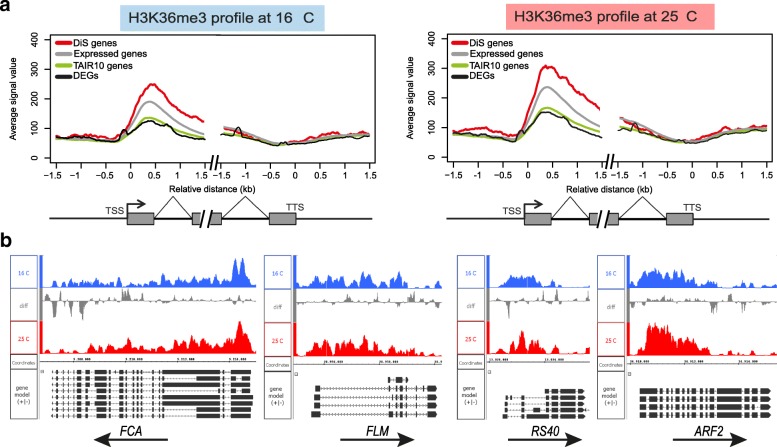
Temperature-induced DiS genes are enriched in H3K36me3. **a** H3K36me3 profile around the TSS and the transcription termination site (TTS), for different classes of genes. **b** H3K36me3 profile for selected genes with temperature-induced DiS events

In mammalian studies, the H3K36me3 mark was found to be enriched in genes undergoing AS and therefore was suggested to play a predominant role in the AS regulation. We hypothesize that the H3K36me3 deposition contributes to the regulation of ambient temperature-induced AS in plants. To investigate the putative role of this mark, we first identified H3K36me3 modified genomic regions in plants growing at 16 °C and in plants that were transferred from 16 °C to 25 °C. Since we observed that most of the DiS events already occur one day after the temperature shift and then remain present (Fig. 1), we selected this time point to investigate the H3K36me3 profile. H3K36me3 chromatin immunoprecipitation sequencing (ChIP-seq) experiments were performed on four biological replicates for each temperature and H3K36me3-marked regions were identified. We found a total of 59,736 regions to be enriched in H3K36me3 (Additional file 4: Table S3). Analyzing the distribution of H3K36me3 along the whole gene body, we observed that H3K36me3 containing histones are prevalent at the beginning of the gene body, with a peak approximately 0.5 kb after the transcription start site (TSS) and predominantly in expressed genes (Fig. 2a). Remarkably, we also observed that DiS genes are enriched in the H3K36me3 mark, while this is not the case for DEGs (Fig. 2a). Around 96% of the DiS genes have an H3K36me3 enriched region in the gene body, while this is only 65% for the DEGs (Additional file 2: Figure S2A). Among the H3K36me3-marked DiS genes, we found key regulators of ambient temperature-sensitive processes, such as the flowering time regulators *FLM*, *MAF2*, and *FCA*, the circadian clock regulators *PRR3* and *PRR7*, and several splicing regulators, such as *RS40* and *SC35-LIKE SPLICING FACTOR 33* (*SCL33*) (Fig. 2b).

The quantitative comparison between the profile obtained before and after the temperature switch (Additional file 4: Table S3) revealed a higher level of H3K36me3 located around 0.5 kb after the TSS in plants exposed at 25 °C (Additional file 2: Figure S2B). For example, we observed higher levels of H3K36me3 at the TSS of the DiS genes *PIN7* and *VIL2* at 25 °C compared with 16 °C (Additional file 4: Table S3).

H3K36me3 regions tend to be more broad at 25 °C compared with 16 °C (Additional file 2: Figure S2B and S3C). For example, we observed an increased in the peak width at *FLM*, *SPL2*, *SCL33*, and at *RUBISCO ACTIVASE* (*RCA*) loci (Additional file 4: Table S3).

A few genes showed a quantitative change in H3K36me3 at the location of a DiS event under these conditions, for example, for the circadian clock genes *PRR3* and *PRR7* (Additional file 2: Figure S3); however, for the majority of cases there was no perfect co-localization.

Thus, overrepresentation of the H3K36me3 histone mark was observed in DiS genes and changes in the mark deposition were observed after increasing temperature, suggesting a role for the mark in the regulation of ambient temperature-induced AS.

### H3K36me3 contributes to regulation of temperature-induced DiS events

**Fig. 3:**
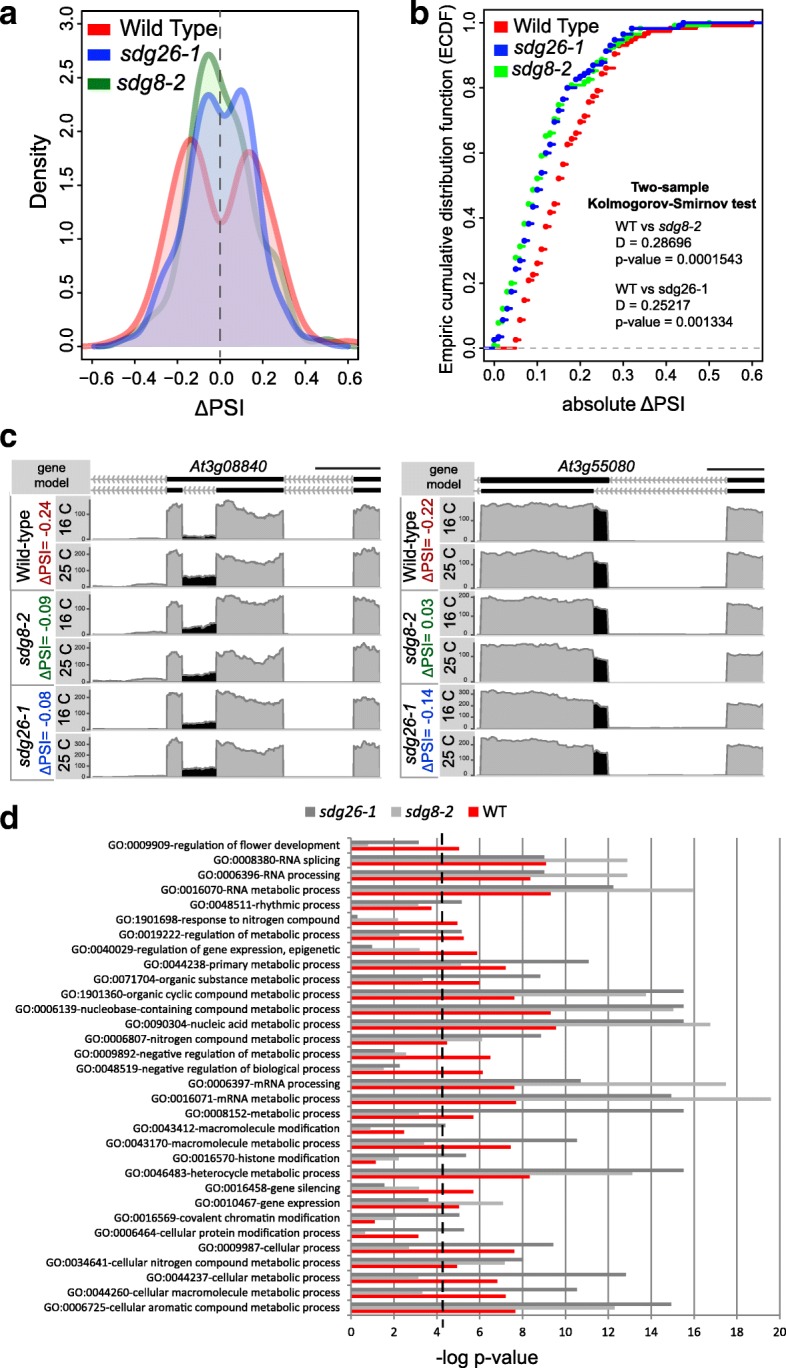
H3K36me3 contributes to regulation of temperature-induced splicing. **a** The *graph* shows distribution of differences in PSI between 16 °C and 25 °C (ΔPSI) of significant DiS events identified in WT and corresponding ΔPSI distributions in *sdg8-2* and *sdg26-1* (Additional file 2: Figure S2C). **b** *Graph* shows plot of empiric cumulative distribution function (ecdf) for absolute ΔPSI of WT, *sdg8-2*, and *sdg26-1* for significant DiS events in the comparison of WT 16 °C and 25 °C. Distribution of ΔPSI are significantly different between WT and *sdg8-2* or *sdg26-1* according to the Kolmogorov–Smirnov test **c** Partial gene model of two examples depicting the DiS event and corresponding plot area showing RNA-seq results. Reads within the AS regions are shown in *black*, other reads are in *gray*. At3g08840 is only significantly DiS in the WT while At3g55080 is significantly DiS in WT as well as in *sdg26-1*. **d** Gene ontology enrichment for genes of which the splicing is affected by ambient temperature conditions in WT, *sdg8-2*, and *sdg26-1*. The histogram includes all overrepresented categories with at least five genes and *p* value < 0.05 for at least one genotype

To elucidate the contribution of H3K36me3 in temperature-induced splicing regulation, we investigated the effect of decreased levels of H3K36me3 on temperature-induced AS. In Arabidopsis, the deposition of H3K36me3 is regulated by the histone methyltransferases SDG8 and SDG26 and, accordingly, levels of H3K36me3 are low in *sdg8-2* and *sdg26-1* mutant plants [44, 45]. We performed an RNA-seq experiment on wild-type (WT) and mutant plants, continuously grown at 16 °C, or one day after a temperature shift to 25 °C. For each genotype, we retrieved DiS events upon the temperature change (Additional file 5: Table S4). Significant differences in temperature-induced DiS events were observed between WT and the H3K36me3 mutants upon the temperature change (Fig. 3). In the *sdg8-2* and *sdg26-1* mutant different types of splicing events are affected by the temperature change (Additional file 2: Figure S4A), indicating that the histone mark is not involved in the regulation of a specific type of event.

Consistent with the contribution of H3K36me3 in temperature-induce splicing, only a minority of DiS genes in WT were also DiS in *sdg8-2* and *sdg26-1* (Additional file 2: Figure S4B), while many events are affected by the decreased levels of H3K36me3 (Additional file 5: Table S4). For example, reduction of H3K36me3 in the mutants disturb temperature-induced AS of flowering time genes, such as *FLM* and *MAF3*, the splicing regulators *SCL33* and *U2AF65B*, and the circadian clock gene *PRR7* (Additional file 5: Table S4).

Because we noticed various cases in which DiS and H3K36me3 deposition were not perfectly co-localized, we investigated if there is a correlation between the location of temperature-dependent H3K36me3 deposition and temperature-dependent AS. We categorized temperature-induced AS events as H3K36me3-dependent if the events are affected by loss of H3K36me3 (significantly DiS in the WT but not in the *sdg8* and *sdg26* mutants) and H3K36me3-independent if the events are not affected by the loss of H3K36me3 (significantly DiS in all the genotypes). H3K36me3-dependent events tend to be located closer to an H3K36me3 region compared with H3K36me3-independent events (Additional file 2: Figure S4C).

We further characterized to which biological processes the genes are associated that undergo DiS in the *sdg8-2* and *sdg26-1* mutants (Fig. 3d). We observed GO categories such as “regulation of flower development” or “gene silencing” to be no longer overrepresented in *sdg8-2* and *sdg26-1*, while GO categories related to metabolic processes appeared higher overrepresented in the mutants.

Finally, we investigated if *sdg8-2* and *sdg26-1* mutants are only affected in temperature-induced AS or generally in the regulation of splicing. We calculated ΔPSI between WT and *sdg8-2* or *sdg26-1* growing at 16 °C. Only a small set of genes show changes in splicing pattern in the mutants (Additional file 6: Table S5). These results highlight a specific function for H3K36me3 in the regulation of high temperature-induced AS.

In conclusion, the higher levels of H3K36me3 in high ambient temperature-induced DiS genes (Fig. 2) and the altered temperature-induced AS for several genes in H3K36me3 mutants (Fig. 3) demonstrate a role of H3K36me3 in regulating temperature-induced AS in Arabidopsis.

### Mutations in H3K36me3 writers, eraser, or readers affected high temperature-induced flowering

**Fig. 4:**
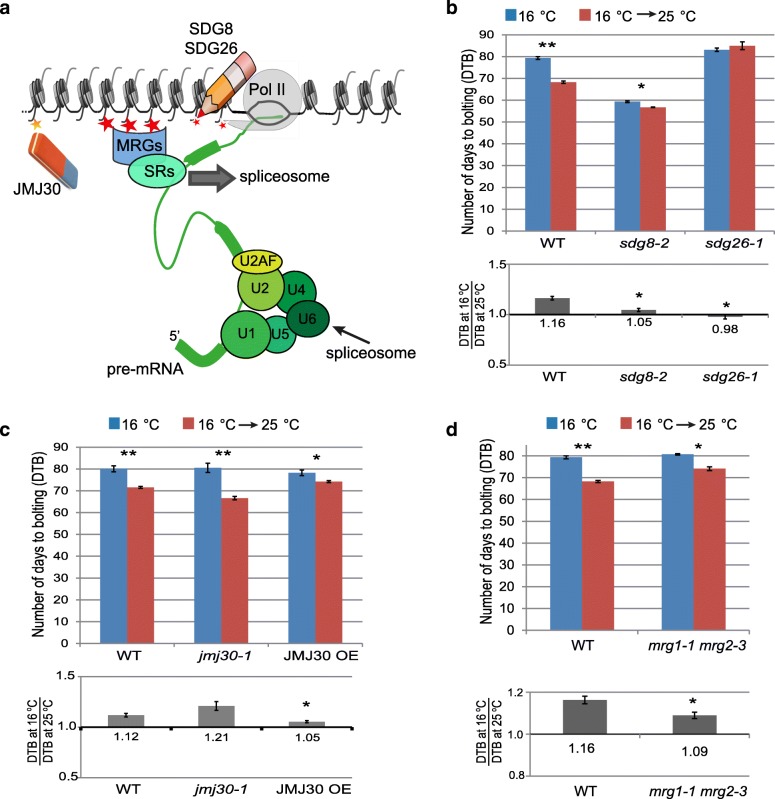
Temperature induced-flowering is compromised in mutant of H3K36me3 writers, eraser, and readers. **a** *Cartoon* illustrates regulation of H3K36me3 and splicing according to the adaptor-complex model in Arabidopsis. H3K36me3 modified histones are bound by an adapter, such as MRG1 and MRG2, that interacts with splicing regulators (SRs), such as PTB. The splicing regulators act in promoting or preventing AS. **b** Flowering time of WT, *sdg8-2*, and *sdg26-1* plants growing at 16 °C constantly or moved to 25 °C after five weeks. In the top *histogram*, flowering time is expressed as numbers of DTB. In the bottom *histogram*, the ratio of DTB between the two conditions is shown. In plants with low levels of H3K36me3, flowering is not promoted by higher temperature. ** indicates significant differences at *p* value < 0.001 while * indicates *p* value < 0.05 according to the Student’s t-test. **c** Flowering time of WT, *jmj30-1*, and *JMJ30-OE* plants is shown as in (**b**). Loss of JMJ30 increases the response to temperature-induced flowering, while an increase in JMJ30 activity causes an opposite effect. **d** Flowering time of WT, *mrg1-1 mrg2-3* plants are shown as in (**b**). *mrg1-1 mrg2-3* plants are less responsive to temperature

In order to investigate the biological relevance of the observed molecular events, we investigated whether a lack of H3K36me3 deposition or lack of proteins recognizing this histone modification affects ambient temperature-controlled flowering. Arabidopsis WT and the H3K36me3 methyltransferase mutants, *sdg8-2* and *sdg26-1*, were grown at 16 °C for five weeks and then maintained at 16 °C or transferred to 25 °C. We quantified temperature response as the ratio in days to bolting (DTB, Fig. 4) or rosette leaf number (RLN, Additional file 2: Figure S5) at low ambient temperatures over the DTB or RLN at high ambient temperatures. Thus, for a genotype that accelerates flowering in higher ambient temperatures, this ratio is higher than 1, as we observed in WT, while for a plant not responding to temperature, this ratio would be 1 [46]. As expected, the increase in temperature accelerated flowering in WT plants (Fig. 4 and Additional file 2: Figure S5). In contrast, *sdg8-2* and *sdg26-1* plants flowered at similar time points (ratio close to 1) at both temperatures (Fig. 4b and Additional file 2: Figure S5A). The significant difference in temperature response between WT plants and the H3K36me3 methyltransferase mutants (Fig. 4b and Additional file 2: Figure S5A) indicates that H3K36me3 is required for temperature-induced flowering.

We also characterized the flowering time response upon a temperature change for the H3K36me3 demethylase mutant *jmj30-1* and for a previously published *JMJ30* overexpression line (*JMJ30-OE*) [47] (Fig. 4c and Additional file 2: Figure S5B). In agreement with the previous experiment we found that *jmj30-1* is more sensitive to the temperature while *JMJ30-OE* is less responsive in temperature-induced flowering (Fig. 4c and Additional file 2: Figure S5B).

In mammals, H3K36me3 has been shown to regulate AS via the adapter-complex model: the protein complex H3K36me3–MRG15–PTB forms a splicing–chromatin co-transcriptional architecture that can bring the splicing machinery in the vicinity of chromatin (Fig. 4a) [32]. In Arabidopsis, the *MRG15* homologs *MORF RELATED GENE 1* (*MRG1*) and *MRG2* are able to bind to H3K36m3 modified histones, as is the case for the human *MRG15* [48]. Moreover, the characterization of *PTB* homologs in Arabidopsis (*PTB1*, *PTB2*, and *PTB3*), reveals their role in the regulation of splicing of flowering time genes, including *FLM* [49]. In the proposed adaptor-complex model [31], the H3K36me3 readers *MRG1* and *MRG2* proteins play a pivotal role. In agreement with this model, we found that *mrg1-1 mrg2-3* mutant plants are less sensitive to temperature-induced flowering than the WT (Fig. 4d and Additional file 2: Figure S5C).

Remarkably, none of the investigated mutant genotypes show strong pleiotropic defects throughout plant development (Additional file 2: Figure S6), in contrast to what was previously observed for core-splicing factor mutants [25, 50–54]. This is in line with our finding that in the H3K36me3 mutants splicing as such is not much affected, but that the defects are specifically for high ambient temperature-induced DiS.

In conclusion, mutations in H3K36me3 writers, an eraser, and readers affect plant response to ambient temperature changes, suggesting a role of this histone mark in plasticity to this environmental cue.

## Discussion

In plants, AS of pre-mRNA is a key mechanism to generate protein diversity and also allows rapid changes in transcript abundance via NMD [55]. In temperature sensing, AS represent a fast and flexible way to confer plasticity in plant development. Our time-series experiments highlight a rapid and rather stable response of changes in splicing upon a temperature change. We found genes belonging to many biological processed to be regulated by temperature-induced AS, such as circadian rhythm and flowering time control, as well as many genes coding for splicing regulators.

The co-transcriptional nature of splicing creates opportunities for cross-regulation between splicing and chromatin landscape. Recent finding indicated that introns are spliced immediately after their transcription [56], indicating the need of a fast recruitment of splicing regulators to the nascent pre-mRNA. In mammals, histone modifications contribute to the regulation of splicing, but whether epigenetic regulation of splicing occurs in plants is unknown. Our results indicate a direct role for histone modifications in AS regulation in plants, highlighting conservation between mammals and plants. However, while in mammals H3K36me3 prevalently regulates ES [32], we found that H3K36me3 is equally involved in the regulation of all type of AS events in plants. Furthermore, in *Arabidopsis* H3K36me3 specifically affects ambient temperature-induced splicing regulation.

While our findings highlight a key role for H3K36me3 in the regulation of ambient temperature-induced splicing, the exact molecular mode of action still remains to be elucidated. Our results do not exclude the kinetic model, neither the adapter-complex model, which have been proposed to explain the linkage between chromatin modifications and AS [28, 31]. Instead, our data suggest that both mechanisms can play a role in H3K36me3 mediated temperature-induced AS. A reduced response to temperature in flowering induction observed in the *mrg1-1 mrg2-3* double mutant suggests that H3K36me3 presence affects splicing outcome at least partially by influencing the recruitment of splicing regulators via a chromatin-binding protein (adapter-complex model). Nevertheless, the preferential location of the H3K36me3 mark in the proximity of H3K36me3 dependent AS events could also point to an effect on transcription speed in support of the kinetic model. In agreement, transcription rate was found to be affected by temperature in Arabidopsis [57]. Additionally, deposition of the Histone 2 variant H2A.Z, which was associated to temperature-mediated flowering time control in Arabidopsis [58], was recently found to affect transcription elongation kinetics and AS in yeast, and especially under destabilizing conditions, such as high temperature [59, 60]. These observations and our results are in favor of the kinetic model. Nevertheless, further research will be essential to characterize the molecular mode(s) of action of H3K36me3 temperature-induced AS regulation in depth.

We show that H3K36me3 plays a key role in the regulation of temperature-induced flowering. Flowering time is a key developmental process, because a proper timing in the switch from the vegetative to the reproductive phase ensures plant fitness. We found that key regulators in the ambient temperature-controlled flowering time pathway are subject to H3K36me3 level dependent AS. However, H3K36me3 may also affect temperature-induced flowering indirectly by affecting splicing of upstream regulators of flowering time genes. For example, the temperature DiS gene *VIL2* regulates the ambient temperature-associated flowering time gene *MAF5* [40]. Alternatively, the effects on ambient-temperature dependent flowering time can be the result of differential gene expression in the various studied H3K36me3-linked mutants.

## Conclusions

AS of pre-mRNA is a key mechanism to generate protein diversity or to regulate transcript isoform abundance and it allows rapid changes in the transcriptome. In temperature sensing, AS represents a fast and flexible way to confer plasticity in plant development. The co-transcriptional nature of splicing creates opportunities for cross-regulation between splicing and chromatin landscape. In mammals, histone modifications contribute to the regulation of splicing. Here we show a direct role for histone modifications in AS regulation in plants. We reveal a key role for H3K36me3 in the control of temperature-induced AS and in temperature-dependent flowering time control in Arabidopsis.

Besides that AS of key regulatory genes confers plasticity, because it is a fast and reversible response to changing environmental conditions, our findings on the role of H3K36me3 in this process open interesting prospective for a memory of temperature mediated AS. It is tempting to speculate that temperature variation is memorized in the chromatin landscape via H3K36me3 deposition, leading to a specific splicing pattern, which allows the memory of environmental conditions during the life cycle and a possible adaptation to a rapidly changing climate.

## Methods

### Plant material and growth conditions

Seeds of knock-out lines, *sdg8-2* Salk_026442 [45], *sdg26-1* Salk_013895 [44], *jmj30-1* (N67787) [47], and the overexpression line JMJ30 (N67797) [47], were obtained from the Nottingham Arabidopsis Stock Center (NASC). Seeds of knock-out line *mrg1-1 mrg2-3* [48] were a gift from Toshito Ito (Temasek Life Sciences Laboratory, Singapore). All plants were grown under short day conditions (8 h light, 16 h dark) on rock-wool at 16 °C for five weeks. Plants were left at the same temperature or moved at 25 °C (6 h after lights on). WT Col-0 plants for the time series RNA-seq experiments were grown in growth cabinets with LED lamps with light at intensity of 200 μmol m-2 s-1 and humidity of 75%. WT and mutant lines for the RNA-seq and flowering time assay were grown in walk-in climate growth chambers with fluorescent lamps with light at intensity of 185 μmol m-2 s-1 and humidity of 65%.

### RNA-seq experiments

Three biological samples were generated for each condition. For each sample, tissue from approximately ten plants was collected 4 h after light on for the time-series experiment and between 4 h and 7 h after light on for the mutant experiment. Using jeweler’s forceps, leaves were removed to obtain SAM enriched tissue. Total RNA was extracted using the InviTrap Spin Plant RNA Mini Kit (REF: 1064100300) from Invitek according to the manufacturer’s protocol. DNAse treatment was performed to remove genomic DNA. DNase I digestion was performed on total RNA using Turbo DNase from Ambion according to the manufacturer’s protocol. RNA integrity was checked on 1% (w/v) agarose gel after DNase I treatment. Samples were prepared for Illumina sequencing using the TruSeq Stranded mRNA Sample Prep kit (REF: 15032613) from Illumina according to the manufacturer’s protocol. Libraries were analyzed on the Bioanalizer and quantified with the qBit before pooling for sequencing on HiSeq2500. Two lanes on a 125pb PE run were used.

### RNA-seq data analysis

RNA-seq reads were mapped against the *Arabidopsis thaliana* genome version TAIR10 (www.arabidopsis.org) using TopHat2 [61]. To improve TAIR10 transcript annotation, reference-based full-length transcript isoform reconstruction was performed for each experiment separately using Cufflinks [62]. Cuffmerge, which is part of the Cufflinks package, was finally used for merging the individual cufflinks results into an overall set of full-length transcripts. Custom python scripts were used for detecting the following type of alternative splicing events: IR, ES, A5, A3, and MXE. MISO software [63] was used to quantify AS-events (PSI values) in each individual sample and in pooled samples that were generated by merging the replicates of each condition. MISO, which also used for the differential splicing analyses, does not have built-in methods for analyzing experimental replicates. As suggested by the authors of MISO, we used the pooled samples for the actual differential splicing test and the individual replicates for filtering AS events based on the following two rules: first, we only considered those AS events that were supported by at least 20 isoform-specific reads in all at least two of the replicates of the conditions under comparison; second, the within-condition PSI differences were required to be smaller than the between-condition PSI differences. Finally, AS events that met the criteria and for which the compare-sample module of MISO returned a Bayes factor of at least 5 were considered significant.

To retrieve differentially expressed genes upon the temperature changes, the number of fragments mapping to TAIR10 annotated genes was determined using HTseq count [64]. Differentially expressed genes were detected using DESeq2 [65].

### GO analysis

GO analysis was performed using the Cytoscape plugin BiNGO [66] using the most recent go-basic ontology file and Arabidopsis gene association file from The Gene Ontology Consortium [67]. Overrepresentation was tested using a hypergeometric statistical test and the Benjamini–Hochberg false discovery rate correction was used. GO category was considered significantly overrepresented if corrected *p* value < 0.05 and at least five genes were present.

### ChIP-seq experiments

Four independent biological replicates for each temperature were generated. For each sample, 0.5 g of plant material was used per biological replicate. Material was collected from plants growing at 16 °C, as well as from plants one day after the plants were moved to 25 °C (5–6 h after lights on). Using jeweler’s forceps, leaves were removed to obtain SAM-enriched tissue. ChIP experiments were performed following a previously published protocol [68]. After the preclearing step, the sample was split, one aliquot was incubated with anti-H3K36me3 antibody (ab9050, abcam) [69, 70], and the other with anti-H3 antibody (ab1791, abcam). Samples were prepared for Illumina sequencing using the ThruPLEX DNA-seq Kit (Cat. No: R400429) from Rubicon Genomics according to the manufacturer’s protocol. Libraries were analyzed on the Bioanlizer and quantified with the qBit before pooling for sequencing on HiSeq2500. In total three lanes on the chip were used and sequenced 50 bp single run (SR).

### ChIP-seq data analysis

FASTQ files were mapped to the Arabidopsis thaliana genome TAIR10, using Bowtie [61], using the default parameters. Reproducibility between biological replicates was assessed using the Pearson correlation for the genome-wide reads distribution at each pair of replicates using DeepTool [71]. We used “multiBamSummary” function with default parameters except for “bin size” that was set to 1 kb and “plotCorrelation” function of deepTools2 in Galaxy (http://deeptools.ie-freiburg.mpg.de/). Regions with very large counts were removed to avoid bias in the Pearson correlation (Additional file 2: Figure S7). To identify H3K36me3 modified nucleosome positions, we used DANPOS2 [72]. We used the “Dpeak” function in DANPOS2 with default parameters, except for the parameter – l (read extension length) that was set to 150 bp, the size of mononucleosomal DNA. Genomic regions were associated with genes if located within the start and the end of the gene using the “intersect” function of BEDTools [73]. The “Profile” function from DANPOS2 was used to retrieve H3K36me3 occupancy profiles around the TSS and the transcription termination site (TTS).

### Flowering time assay

For flowering time analyses, plants were randomly arranged in trays and grown under white light (185 μmol m-2 s-1) in short day conditions (8 h light, 16 h dark) in walk-in climate chambers with humidity of 65%. Trays were rearranged every two days and nutrient was supplied by sub-irrigation. After five weeks of growth at 16 °C, half of the plants were moved to the 25 °C growth chamber. Flowering time was quantified by determining the time until the macroscopic appearance of the first flower bud (DTB), screening was done every two days and by counting RLN after bolting of all plants in the tray. The experiment was performed in three biological replicates with 15 plants in each replicate for each genotype/condition. Data are shown as average of ratios between the replicates. Student’s t-tests were calculated with GraphPad QuickCalcs.

## Additional files

Additional file 1: Table S1. List of significant (see Methods) DiS events in at least one 25 °C time point (day 1, 3, or 5) compared to the 16 °C. (XLSX 75 kb)

Additional file 2: Supplementary Figure S1-S7. Temperature induced AS events. **Figure S2.** H3K36me3 dynamics upon the temperature change. **Figure S3.** Quantitative change in H3K36me3 (ChIP-seq output) at the same location of a DiS event (RNA-seq output) for the circadian clock genes PRR3 and PRR7. **Figure S4.** Temperature-induced AS in sdg8-2 and sdg26-1. **Figure S5.** Changes in H3K36me3 levels and mrg1 mrg2 affect temperature induction of flowering. **Figure S6.** Changes in H3K36me3 levels do not cause pleotropic effects on plant development. **Figure S7.** Pearson correlation of ChIP-seq experiments. (PDF 1186 kb)

Additional file 3: Table S2. DEGs between 16 °C and 25 °C day 1 (sheet1), 16 °C and 25 °C day 3 (sheet2), 16 °C and 25 °C day 5 (sheet3). DEGs are defined as genes with a change in expression of log2 Fold Change |1| and adjusted *p* value according to the BH method for controlling false discovery < 0.05. (XLSX 518 kb)

Additional file 4: Table S3. H3K36me3 modified regions at 16 °C or 25 °C day 1 and quantitative comparison obtained with DANPOS2. (XLSX 15623 kb)

Additional file 5: Table S4. List of significant (see “Methods”) DiS events between 16 °C and 25 °C day 1 in at least one mutant genotype. (XLSX 5555 kb)

Additional file 6: Table S5. List of significant (see “Methods”) DiS events between WT and *sdg8-2* and events between WT and *sdg26-1* at 16 °C. (XLSX 17 kb)
