# Supplementary material for: Histone H3 lysine 36 methylation affects temperature-induced alternative splicing and flowering in plants
Source: Genome Biol. 2017 Jun 1;18:102. doi: 10.1186/s13059-017-1235-x (PMC5452352; doi:10.1186/s13059-017-1235-x)
Supplement: Supplementary file 2 — Temperature induced AS events. Figure S2. H3K36me3 dynamics upon the temperature change. Figure S3. Quantitative change in H3K36me3 (ChIP-seq output) at the same location of a DiS event (RNA-seq output) for the circadian clock genes PRR3 and PRR7. Figure S4. Temperature-induced AS in sdg8-2 and sdg26-1. Figure S5. Changes in H3K36me3 levels and mrg1 mrg2 affect temperature induction of flowering. Figure S6. Changes in H3K36me3 levels do not cause pleotropic effects on plant development. Figure S7. Pearson correlation of ChIP-seq experiments. (PDF 1186 kb) [file 13059_2017_1235_MOESM2_ESM.pdf]

Supplementary figures:

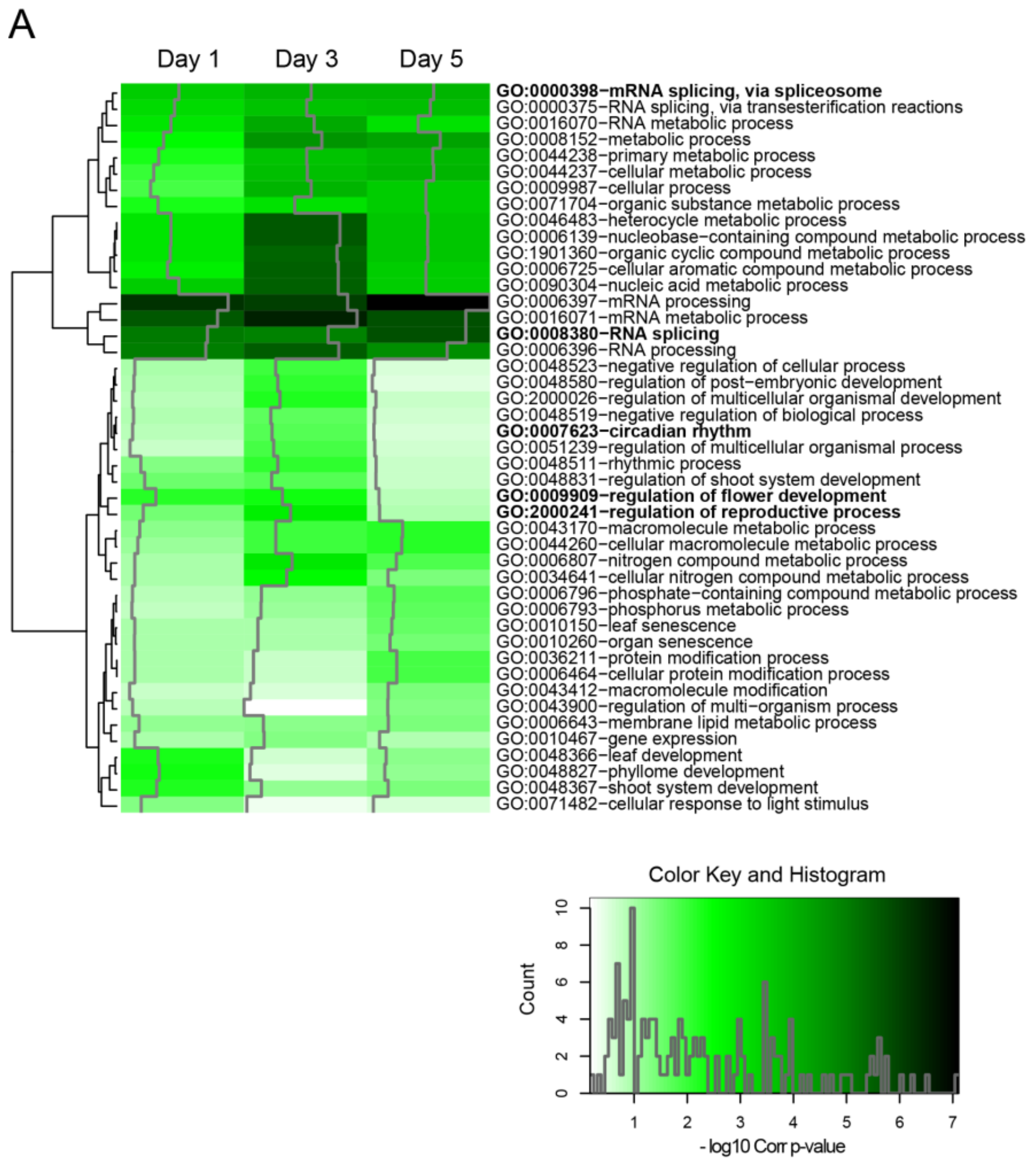

Figure S1. Temperature induced AS events. A. Biological processes affected by temperature-induced differential splicing. Gene ontology enrichment for genes affected by temperature-induced splicing. The heat map includes all overrepresented categories with at least 5 genes and p-value < 0.05 for at least one time point.

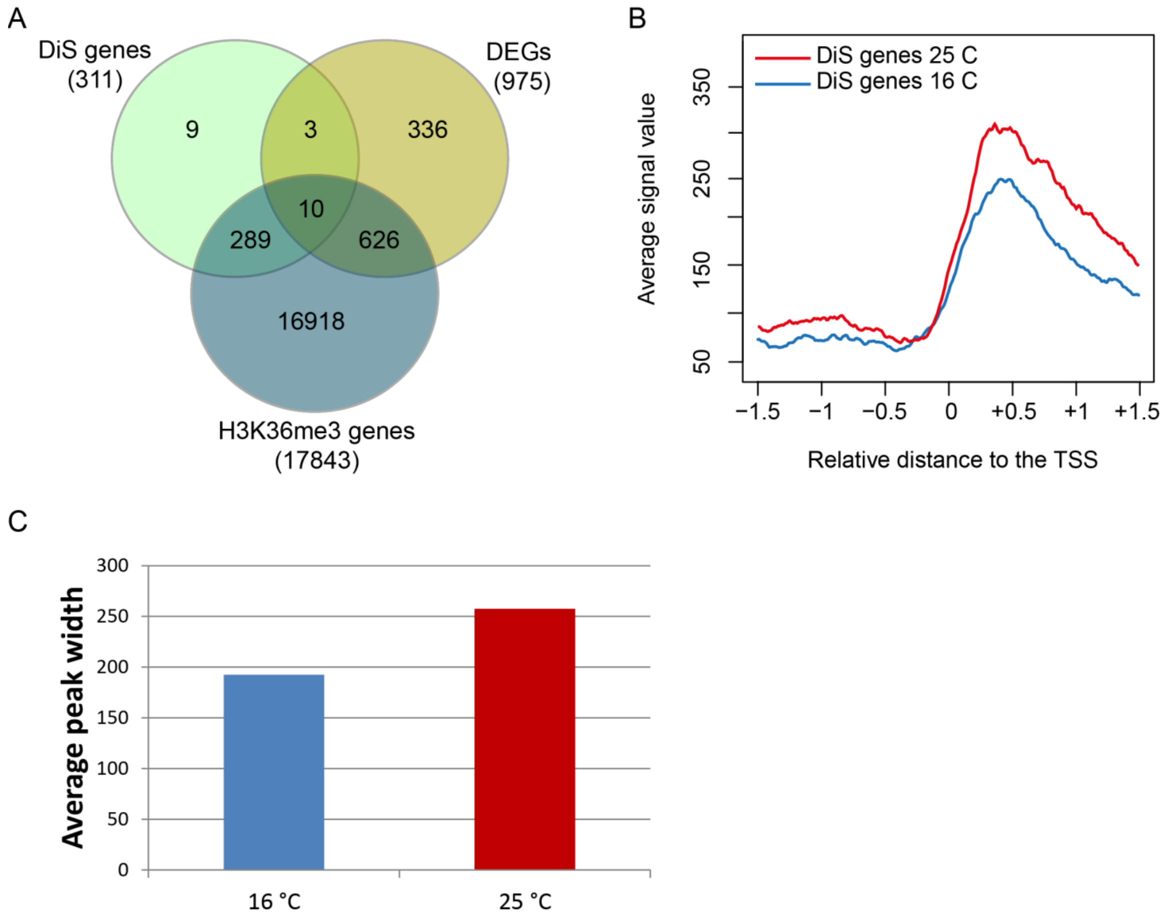

Figure S2. H3K36me3 dynamics upon the temperature change. **A.** Venn diagram shows the proportion of H3K36me3 marked genes that are differentially spliced (DiS) and differentially expressed genes (DEGs) one day after the temperature change. DEGs are defined as genes with a change in expression of log2 Fold Change |1| and adjusted p-value according to the BH method for controlling false discovery (p-adj) < 0.05. **B**. H3K36me3 abundance is plotted around the TSS. An increase in the deposition of H3K36me3 is observed upon a change in temperature. **C.** Peak width average for H3K36me3 modified genomic regions at 16 °C and 25 °C. H3K36me3 modified regions are more broad at the higher temperature.

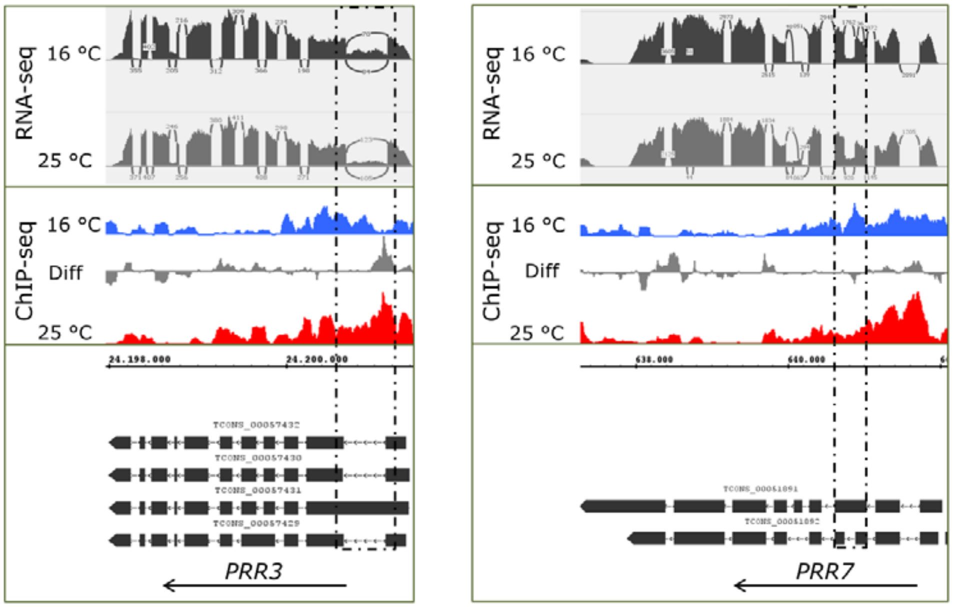

Figure S3. Quantitative change in H3K36me3 (ChIP-seq output) at the same location of a DiS event (RNA-seq output) for the circadian clock genes *PRR3* and *PRR7.*

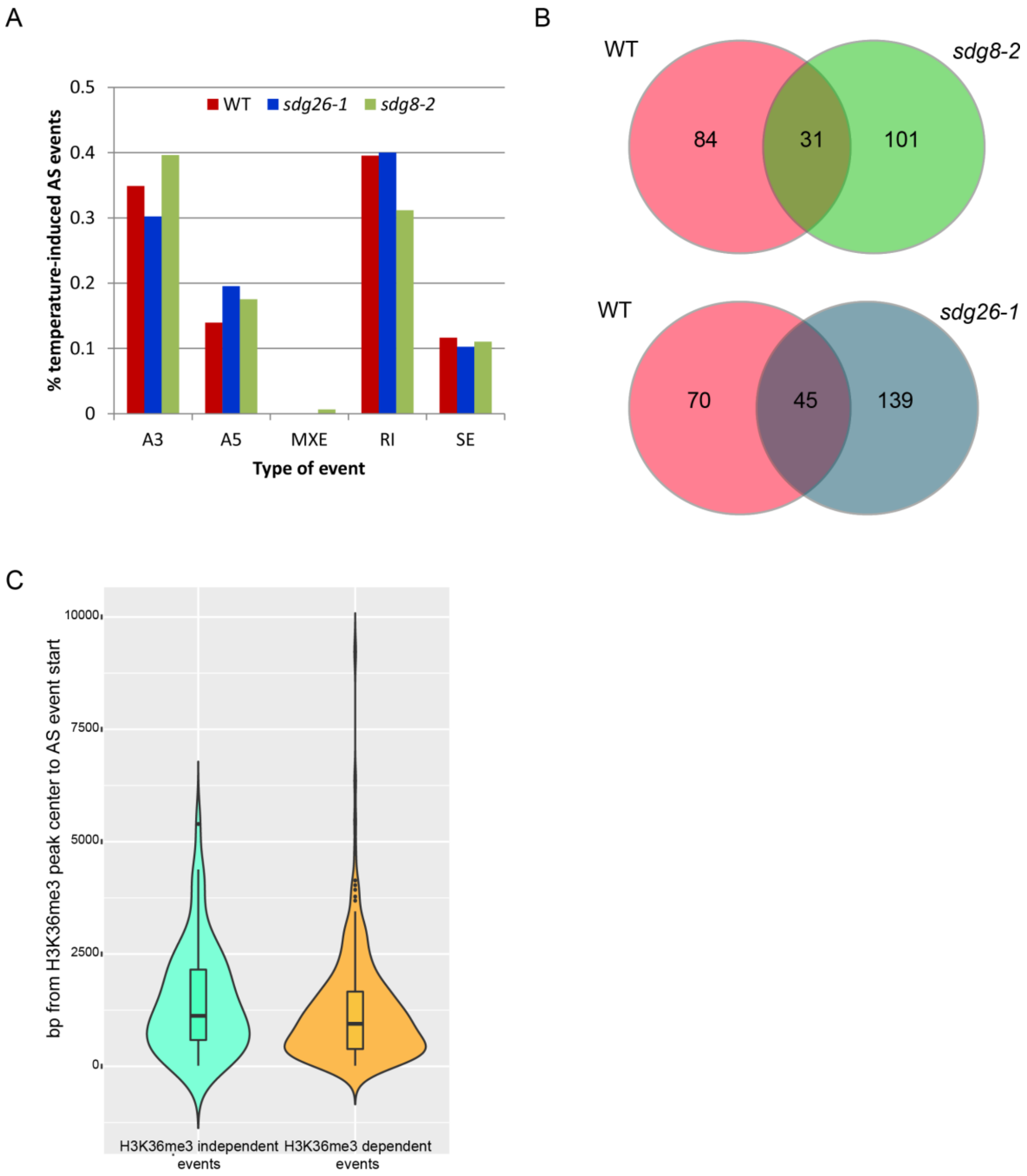

Figure S4. **Temperature-induced AS in *sdg8-2* and *sdg26-1*. A**. Percentage of temperature-induced AS events between the different categories for each genotype. RI, intron retention; MXE, mutually exclusive exon; ES, exon skipping; A5, alternative 5' site; A3, alternative 3' site **B**. Venn diagrams show significant DiS genes in WT vs *sdg8-2* and WT vs *sdg26-1*. **C.** Violin plots showing the absolute distance (bp) from the AS start site to the H3K36me3 enriched region for H3K36me3-independent AS event (events not affected in the *sdg8* and *sdg26* mutants) and H3K36me3-dependent AS event (events that are disturbed in the *sdg8* and *sdg26* mutants). AS events for each genotype are reported in Table S4. Overall the H3K36me3 region tend to be closer to the AS site for H3K36me3-dependent AS event than to the H3K36me3-independent events.

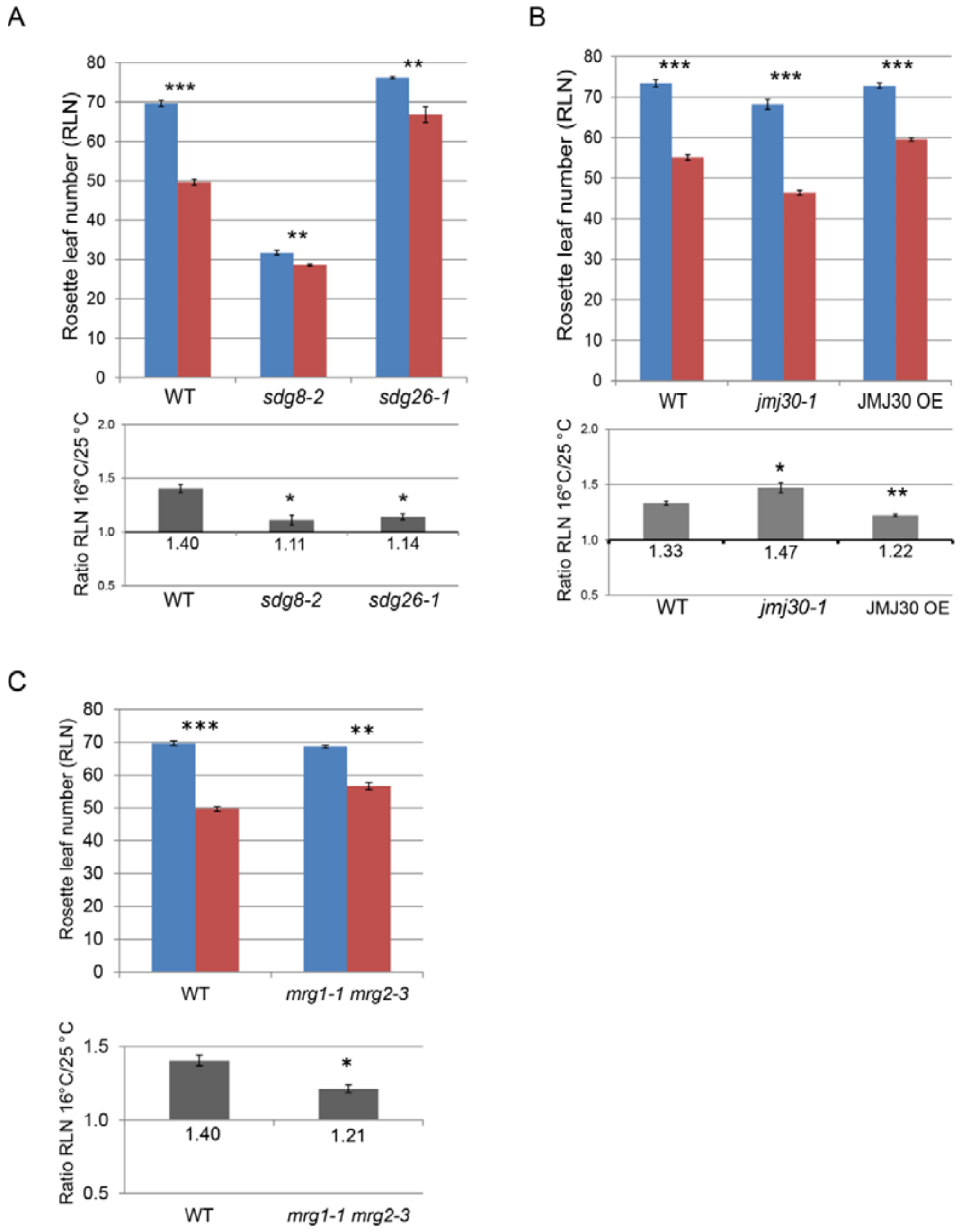

Figure S5. Changes in H3K36me3 levels and *mrg1 mrg2* affect temperature induction of flowering. A. Flowering time of wild-type, *sdg8-2* and *sdg26-1* plants growing at 16°C constantly (blue) or moved to 25°C after 5 weeks of growth at 16°C (red). In the top histogram flowering time is expressed as rosette leaf number (RLN). In the bottom histogram is shown the ratio of RLN between the two conditions. **B.** Flowering time of wild-type, *jmj30-1* and *JMJ30-OE* plants is shown as in A. **C.** Flowering time of wild-type, *mrg1-1 mrg2-3* plants is shown as in A.

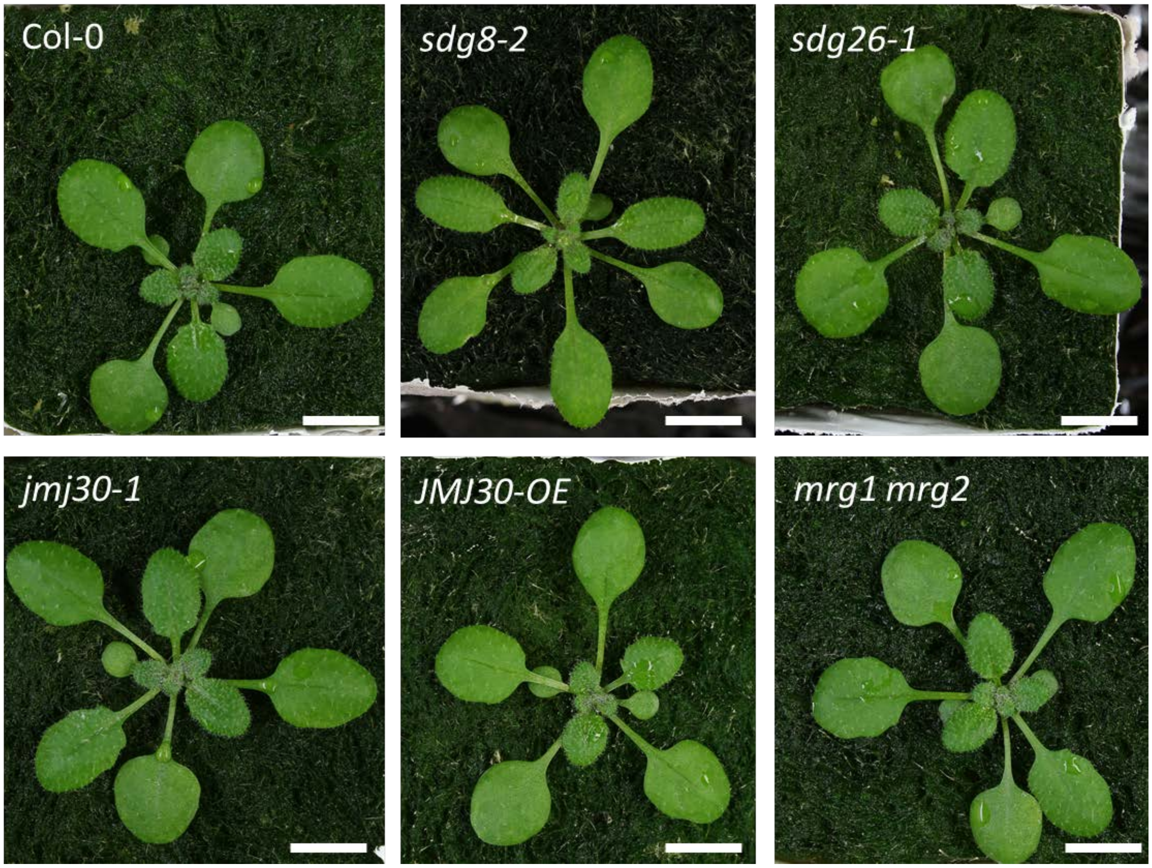

Figure S6. Changes in H3K36me3 levels do not cause pleotropic effects on plant development. Pictures of three week old wild-type and mutant plants used in the study, no visible changes in plant morphology are observed. Note that in the early flowering *sdg8-2* mutant background the inflorescence is already visible.

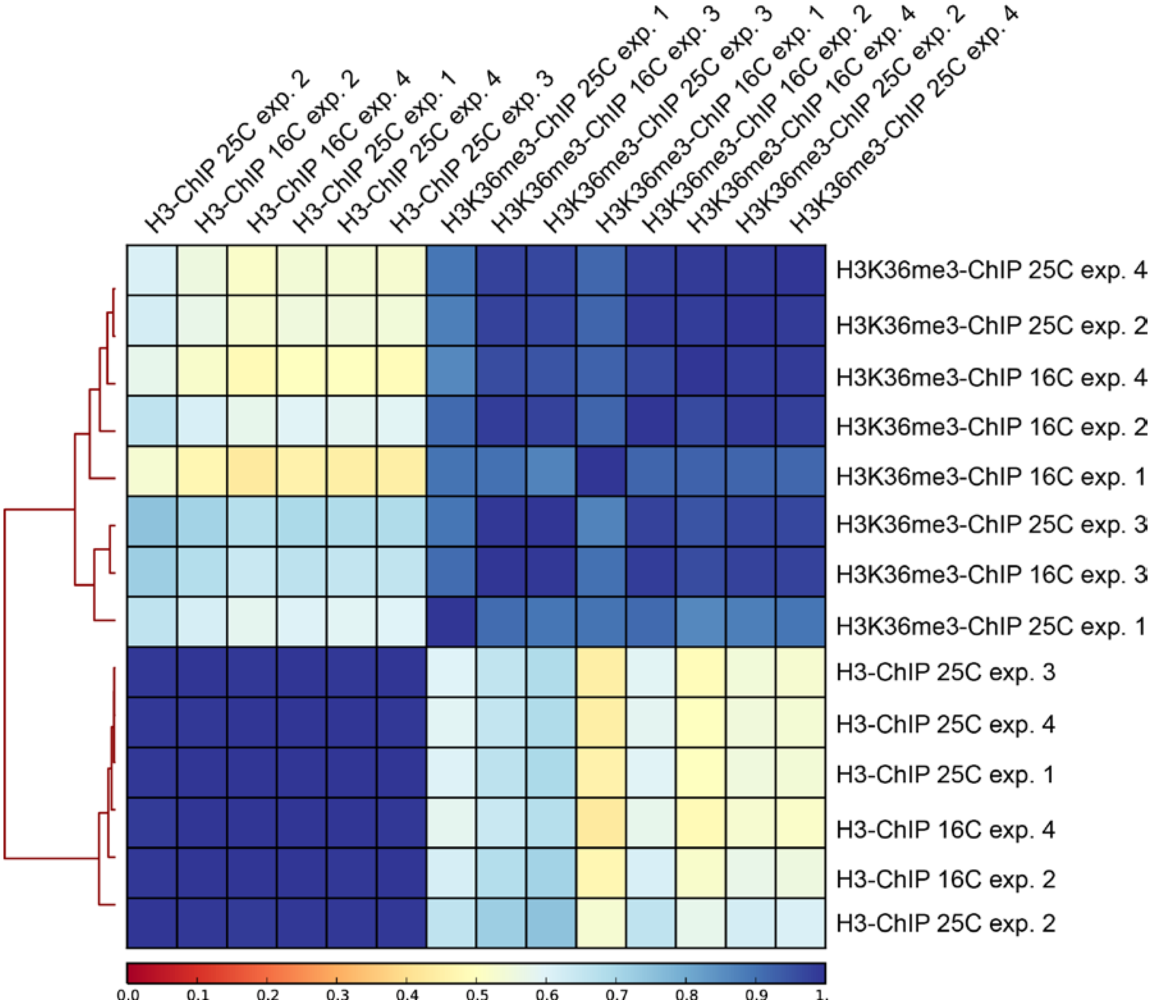

Figure S7. Pearson correlation of ChIP-seq experiments.
